# Reproductive characteristics in female Swedish moose (*Alces alces*), with emphasis on puberty, timing of oestrus, and mating

**DOI:** 10.1186/1751-0147-56-23

**Published:** 2014-04-16

**Authors:** Jonas Malmsten, Lennart Söderquist, Carl-Gustaf Thulin, Dolores Gavier Widén, Lisa Yon, Michael R Hutchings, Anne-Marie Dalin

**Affiliations:** 1 Department of Clinical Sciences, Division of Reproduction, Swedish University of Agricultural Sciences, Box 7054, SE-750 07 Uppsala, Sweden; 2 Department of Pathology and Wildlife Diseases, National Veterinary Institute, SE-751 89 Uppsala, Sweden; 3 Department of Wildlife, Fish, and Environmental Studies, Swedish University of Agricultural Sciences, SE-901 83 Umeå, Sweden; 4 Department of Biomedical Sciences and Veterinary Public Health, Swedish University of Agricultural Sciences, Box 7028, Uppsala, Sweden; 5 Faculty of Medicine and Health Sciences, School of Veterinary Medicine and Science, Sutton Bonington Campus, University of Nottingham, Nottingham LE12 5RD, United Kingdom; 6 Animal and Veterinary Sciences, SRUC, Roslin Institute Building, Easter Bush, Midlothian EH25 9RG, United Kingdom

**Keywords:** Reproduction, Moose, Puberty, Ovulation rate, Mating

## Abstract

**Background:**

The moose (*Alces alces*) is an intensively managed keystone species in Fennoscandia. Several aspects of reproduction in moose have not been fully elucidated, including puberty, timing of mating and oestrus, and the length of the oestrus period. These aspects are relevant for an adaptive management of moose with respect to harvest, population size, demography and environmental conditions. Therefore, an investigation of female moose reproduction was conducted during the moose-hunting period in southern Sweden from 2008 to 2011.

**Results:**

A total of 250 reproductive organs and information on carcass weight and age was collected from four different hunting areas (provinces of Öland, Småland, Södermanland, and Västergötland) in southern Sweden. The results showed that puberty in female moose varied with carcass weight, age, and time of season. The period for oestrous/mating lasted from about mid September to the beginning of November.

**Conclusions:**

The oestrus period (predominantly for heifers) is longer than previously reported and was not finished when the hunting period started. Sampling the uterine cervix to detect spermatozoa was a useful method to determine if mating had occurred. To avoid hunting of moose during oestrus, we suggest that the hunting period should be postponed by at least 14 days in southern Sweden.

## Background

In Fennoscandia, moose (*Alces alces*) are regarded as the most important game species, and considered a symbolic animal of the fauna where almost 200,000 moose are harvested annually in Finland [1], Norway [2], and Sweden [3]. The moose populations are thus intensively managed, and also well studied since the middle of the 20^th^ century [4]. As with all managed game species, knowledge about basic reproductive characteristics and performance is important for an adaptive management strategy. In addition, changes in the surrounding environment and climate may also influence the reproductive capacity in the moose [5].

Fennoscandian moose populations tend to have a skewed age distribution, in which young females account for a large part of the population [6,7]. Thus, age and weight at puberty consequently have an important impact on the proportion of pregnant females in a population and, therefore, population dynamics. In several previous Fennoscandian studies, sexual maturity rather than puberty has been documented [5,8,9]. Sexual maturity is in these studies was defined as the age at which a female has produced its first offspring, whereas puberty normally is defined as when a female has passed her first oestrus and ovulated. The determination of puberty and its association with age and body weight, however, provides more important information on the reproductive development of a female than when a female has first produced offspring. Saether and Haagenrud [10] investigated weight at puberty in Norwegian moose, but the age distribution was not studied, since the only category included in the study of puberty was yearlings (aged 1–2 years). It appears there is no information on both weight and age at puberty in Fennoscandian moose.

Moose are seasonal polyoestral mammals and mating takes place during the autumn. The seasonal oestrous period has been reported to occur during late September and early October [5,10,11] but varied within regions, and between years. During oestrus, one or two dominant follicles (rarely but occasionally three) reach maturation and ovulation takes place.

The timing of oestrus and ovulation during the reproductive season in Fennoscandian moose has been determined in a number of reports dating back to the 1960s and 1970s [4,11-13], but the methods used, and results have varied. A report in Norwegian moose [5] where hunter-collected, formalin-fixed ovaries were used showed substantial spatial and temporal variations in the timing of ovulation for moose of different ages. In moose, one or two follicles ovulate (rarely three), and the number is associated with female age and nutritional status, as older females in good body condition usually ovulate at a higher frequency [4,5,9,14]. Nutritional status is dependent on population density and forage availability, and is therefore also linked to management strategies and to variations in climate. Based on observations of radio-collared animals over several years, the mean number of calves per young (up to 5 years of age), and old (>12 years of age) females was determined to be lower than in mid-aged females (5–12 years of age [15]).

Reproduction can be assessed in different ways, and hunter observations (number of observed calves per observed female) is commonly recorded in Fennoscandia [16,17]. Field observations of radio-collared (VHF or GPS) females similarly locate animals and record the number of calves observed with each female. A third way of studying reproduction is the examination of reproductive organs from hunted animals; this provides information on the reproductive capability in a harvested population, provided that the harvested animals to some extent reflect the rest of the population.

In moose, it is not known whether ovulation occurs during oestrus, or immediately after the end of standing oestrus. In dairy cattle, ovulation occurs approximately 12–16 hours after the end of standing oestrus [18-20], and in captive red deer (*Cervus elaphus*) it is reported to occur up to 40 hours after the end of standing oestrus [21]. When examining reproductive organs during the period from ovulation to detectable pregnancy, it is not possible to macroscopically evaluate if mating has taken place or not. However, information on the possible occurrence of mating can aid in assessing the level of interactions between bulls and cows i.e. whether or not the bull/cow ratio and bull age distribution is optimal. If the proportion of mated females is low, it is possible that there are factors which are disturbing mating activities.

The aims of the current study were, a) to assess weight and age at puberty in Swedish moose heifers, b) to assess oestrus in relation to the hunting period in Sweden, c) to investigate ovulation patterns in relation to age and body weight, and d) to investigate the proportion of mated females (before detectable pregnancy).

## Methods

**Figure 1:**
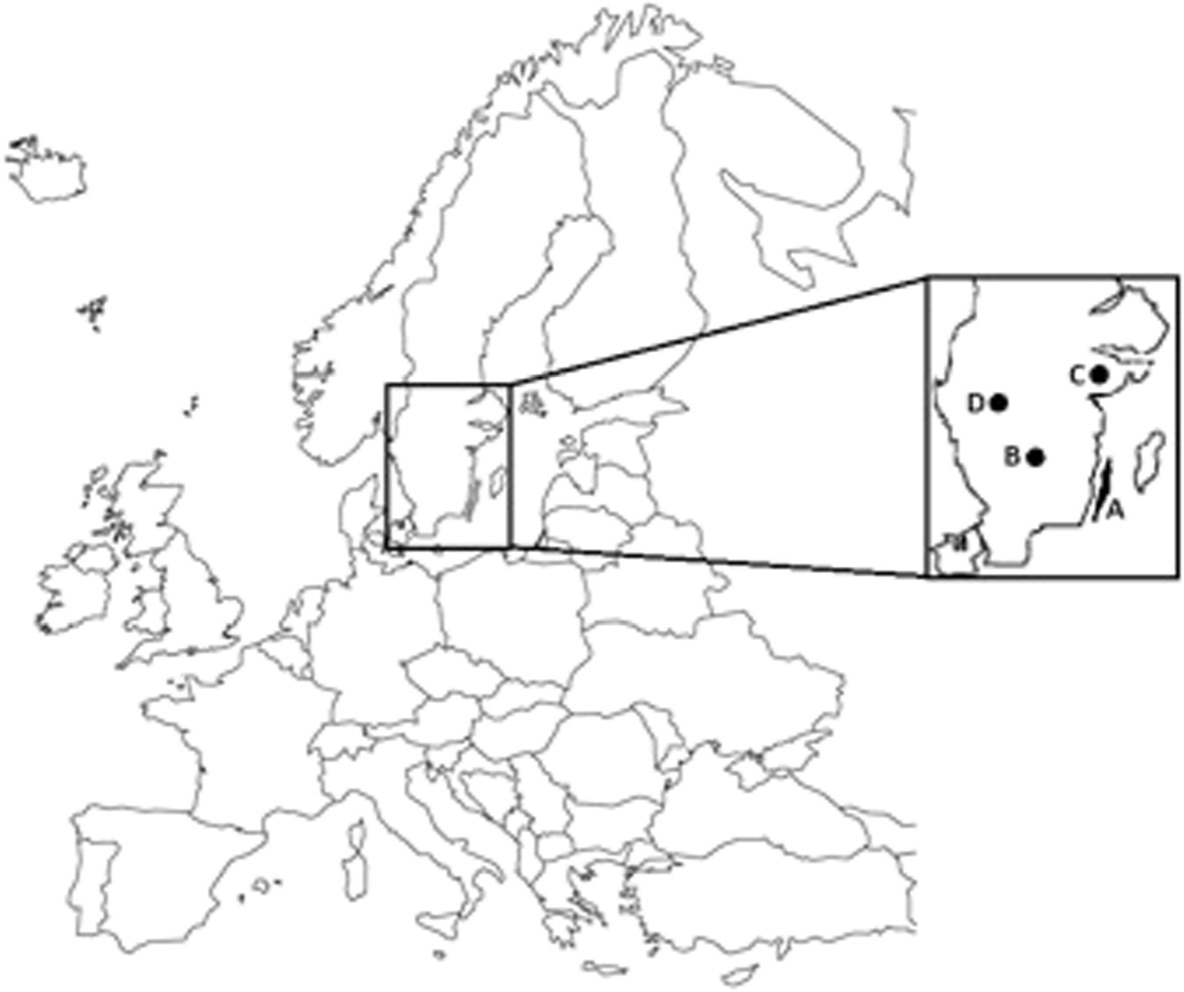
**Map of Europe and southern Sweden (enlarged) with the four sampling locations of genital organs from female moose (*Alces alces*).**

The study area consisted of four provinces in southern Sweden (A: Öland, B: Småland, C: Södermanland and D: Västergötland), ranging from N 56° 55.450′, E 14° 45.056′ to N 59° 5.323′, E 17° 22.600′ (Figure 1). From 2008 to 2011, reproductive organs (ovaries and uteri), and mandibles (for age determination) were collected from hunter-harvested female moose. In area A, material was collected also in 2007. In three areas (B, C, and D; Figure 1), hunting started on the second Monday of October each year (2007: Oct 8, 2008: Oct 13, 2009: Oct 12, 2010: Oct 11, 2011: Oct 10; all dates defined as day zero), in accordance with Swedish hunting legislations. In area A, hunting commenced on the last Saturday of October, based on a mutual agreement among local hunters. Individual identification numbers were assigned to each moose, and subsequent samples, and time and date of harvest were noted. Each sampling year, a temporary field laboratory was used in the hunting areas during the first four to seven days. Trained field personnel collected the material at the site of harvest, and stored it in a cool bag (at approximately +8**°**C) during the transport to the field laboratory for macroscopic investigation and sampling. When no field laboratory was available, samples were collected by the hunters, frozen, and transported to the Swedish University of Agricultural Sciences in Uppsala, Sweden, where they were analysed using the same protocol as used in the field laboratory. Individual moose carcass weight (the weight of the carcass without skin, head, blood, metapodials and internal organs, [22]) was recorded by the hunters and reported to the research team.

Upon arrival at the laboratory, reproductive organs were macroscopically examined according to a standard protocol developed for this study (Table 1). Uterine specimens and ovaries were placed in 10% formalin for later examination. The first molar of the lower jaw was sectioned and cementum layers counted as described by Wolfe [23] for age determination of each adult (age > 1 year). The animals were then divided into three age classes (1–5, >5-12, and >12 years of age) according to Ericsson *et al.*[15].

**Table 1:** **Examination of female reproductive organs from hunter-harvested Swedish moose ( *Alces alces *)**

**Structure**	**Substructure**	**Count**	**Measurement**	**Other**
***Uterus***			L^1^, WGHT^2^,	Content (embryos/fetuses, fetal membranes, fluid,), Appearance: presence of enlarged blood vessels in the cut surface between the broad ligament and the uterus.
	Caruncles	Number		Appearance: prominent or non-prominent
	Mucosa			Appearance: colour, texture
	Embryos	Number	CRL^3^, WGHT	
	Fetuses	Number	CRL^3^, WGHT	
**Ovaries**	*Corpus luteum*	Number	L^1^, W^4^, H^5^, WGHT	
	Follicles	Number	Diameter (mm)	
	*Corpus albicans*	Number		
***Cervix***				Content (spermatozoa)

^1^L = length (cm).

^2^WGHT = weight (gram).

^3^CRL = crown-rump length (cm).

^4^W = width (cm).

^5^H = height (cm).

The examined females were classified in nine different categories (1–9) according to their reproductive status, following specific criteria (see Table 2). Reproductive terminology from domestic cattle was used, where “heifer” describes a female not having given birth regardless of age. The definition of puberty used in the present study was a female that had had her first oestrus in life and ovulated. A “cow” was defined as a female that had been pregnant on one or more occasions, i.e. had one or more corpora albicantia (pigmented scar from a corpus luteum of pregnancy) in the ovaries, as well as enlarged blood vessels in the cut surface between the mesometric ligament (*Ligamentum mesometrium*) and the uterus. All fixed ovaries from cows were sectioned into 1–2 mm slices that were macroscopically examined for presence and number of corpora albicantia. The same procedure was performed at random in some samples from heifers for verification of the method, and where the categorization of the female was uncertain based on the appearance of the uterus or the determined age of the female.

**Table 2:** Criteria for categorization of reproductive stage of harvested female moose from southern Sweden

**Category**	**Description and criteria for the different categories based on appearance and size of structures in the ovaries and the uterus.**
**1**	Pre-pubertal heifer: *small uterus and ovaries, no corpus luteum, i.e. no sign of previous ovulation.*
**2**	Heifer, pubertal, in first oestrus: *large mature follicle (>10 mm), close to ovulation, or recently ovulated (corpus luteum <10 mm, passed puberty).*
**3**	Heifer, pubertal, passed first oestrus: corpus luteum ≥10 mm (in first dioestrus*).
**4**	Heifer, pregnant (>2 weeks): *presence of embryo or fetus.*
**5**	Cow, in oestrus or recently passed first oestrus of the season*: presence of mature follicle (>10 mm) or newly developed corpus luteum (<10 mm).*
**6**	Cow, passed first oestrus of season: *large corpus luteum (≥10 mm).*
**7**	Cow, pregnant (>2 weeks): *presence of embryo or fetus.*
**8**	Cow, returned to oestrus: *Presence of regressed corpus luteum, together with a mature follicle (>10 mm), or a new corpus luteum.*
**9**	Cow in anoestrus: *no signs of large mature follicles (>10 mm) or ovulation (no fresh corpus luteum) during the present season.*

*luteal stage of the oestrus cycle where the progesterone production of the corpus luteum reaches its peak.

Duration of pregnancy was estimated by measuring crown-rump lengths (CRL) of the embryos and comparing the information with previous findings in moose, and corresponding information reported in cattle [4,24].

To determine if mating had occurred, samples were taken with a scalpel from the mucosal folds of the uterine cervix of heifers and cows, then smeared on a glass slide and examined under a light microscope (250-400×) for presence of spermatozoa. This sampling was done in animals that were determined to be in oestrus (with mature follicle) or had passed oestrus (corpus luteum present). The procedure was not performed in pregnant animals (with embryo/foetus present in the uterus).

In order to determine the effect of carcass weight on puberty, a logistical regression model was used, with carcass weight for females in categories 1–4 as the explanatory variable and female reproductive category as the response variable. Another logistical bivariate regression model was used to predict the probability of having passed oestrus in four different weight categories of heifers. Day zero was set as the opening day of the moose-hunting period each year, and date of harvest was set as the number of days related to day zero. The effects of harvest date, sampling area and sampling year on the different female reproductive categories were determined using a logistic regression model created in R [25], with female reproductive category as the response variable. Data from females without any ovarian activity (no follicles or corpora lutea; categories 1 and 9) was not included for determination of the timing of oestrus. Furthermore, the effect of age on ovulation rates for heifers and cows, respectively, was determined using logistic regression in a general additive model (GAM).

All sampling of hunter-harvested moose were conducted with ethical permission (no. C194/7) issued by Uppsala Ethical Review Committee on Animal Experiments.

## Results

In total, 250 female yearling (>1 year of age) and adult (>2 years of age) moose aged between 1.5 and 18.5 years old (mean age 4.3 years) were sampled during 2007–2011. The period of sampling ranged from day zero (the opening day of the hunting period) today 103. For 13 samples, harvest date was not recorded, but for the remaining 237 samples, the majority (66.7%, n = 158) were collected during the first week of the hunting period, and 93.7% (n = 222) during the first month. Of all collected samples, 195 (78.0%) were collected when field laboratories were present, the remaining (n = 55, 22.0%) were sampled by hunters later and thus frozen prior to investigation. In the animals for which age was determined (n = 235), 75.3% (n = 177) were between 1.5 and 4.5 years old. Mid-aged (5–12 years of age), and old-aged (>12 years of age) females accounted for 16.6% (n = 39), and 8.1% (n = 19), respectively. Age was not determined in 15 animals, due to failure to retrieve lower jaws. Carcass weights were recorded in 74.8% (n = 187) of the harvested moose, where the mean weight was 151.5 kg (range 72–220).

**Figure 2:**
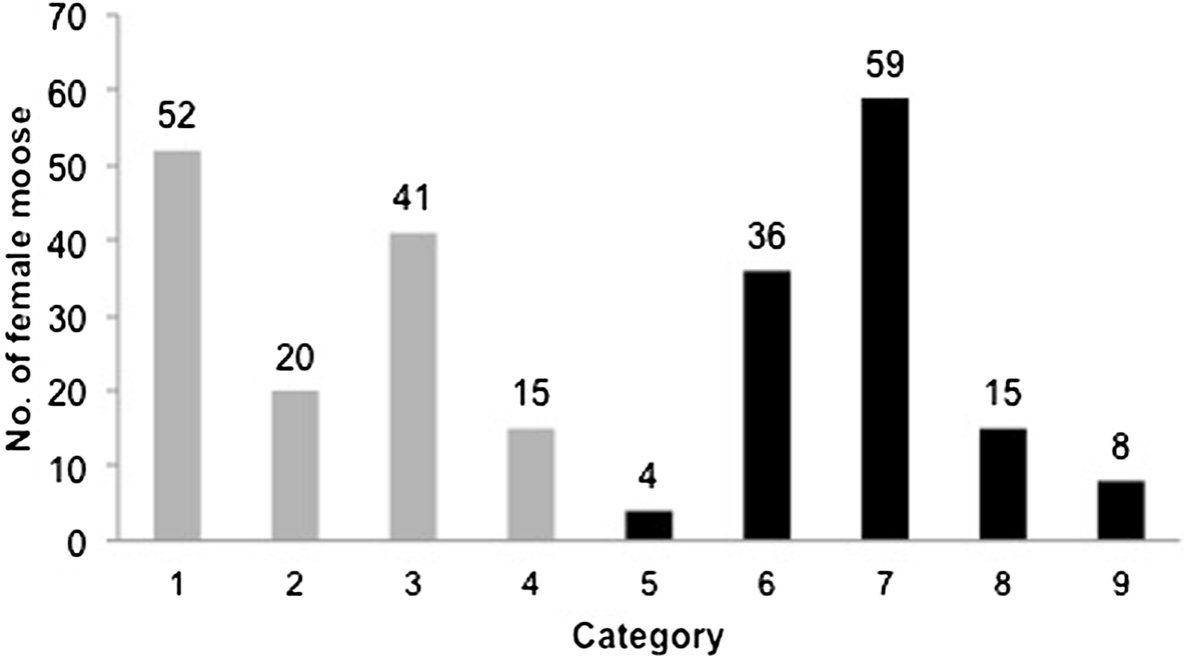
Number of females in the different categories (1–9) based on the examination of reproductive organs from moose collected during the hunting period (October to January) in southern Sweden from 2007 to 2011 (Grey bar = heifers, black bar = cows).

Heifers (Category 1, 2, 3, and 4), accounted for 51.2% (128/250) of all samples, of which 40.6% (52/128) were pre-pubertal heifers (Category 1). Of the heifers 11.7% (n = 15) of all heifers) were pregnant (Figure 2). The remaining samples were from cows (48.8%, 122/250) that had experienced one or more pregnancies in previous years (category 5, 6, 7, 8 and 9). Of the cows, 6.6% (n = 8) were anoestral (Category 9), and of the remaining cows (114 individuals) the majority (94.8%, n = 110) had passed their first oestrus of the season; of these, 40 individuals were pregnant (Figure 2).

In the cows, the mean number of corpora albicantia in the ovaries was 3.85 (range 1–16); the highest number was recorded in a cow aged 17.5 years. There was a positive correlation between age and number of corpora albicantia (*P <* 0.01, adj. R^2^-value 0.546). In the ovaries from heifers that had been randomly selected and examined, no corpora albicantia were found.

**Figure 3:**
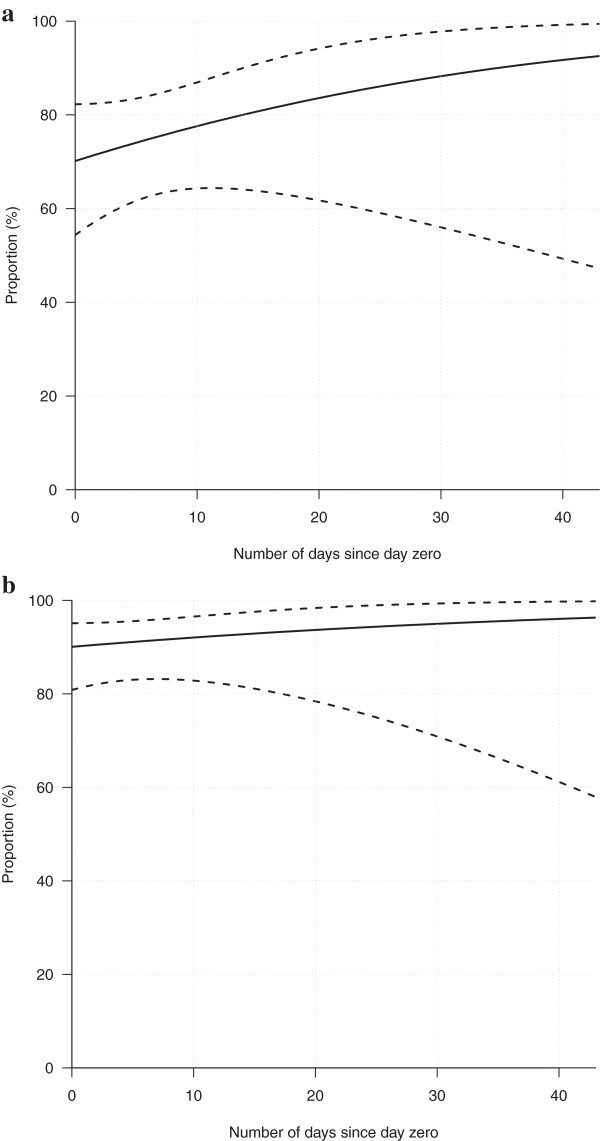
Logistical regression model (95% confidence intervals) with the proportion of heifers (a) and cows (b) having passed oestrus in relation to day zero of the hunting period.

The proportion of heifers and cows (with 95% confidence intervals) determined to have passed oestrus and ovulated (corpus luteum present in the ovaries) at the time of harvest is illustrated in Figure 3a (heifers) and 3b (cows). In pubertal heifers, 68.2% had passed oestrus on day zero, and the corresponding proportion for cows was 90.1%. Thirty days after day zero, 11% of the heifers were still prepubertal (had not passed oestrus), and five per cent of the cows were still anoestral (had not ovulated, although ovarian activity was detected). No significant effect of sampling year or area was observed in the logistical regression model.

The microscopic examination of smears from the cervix was performed in 53 of 61 heifers from categories 2 and 3. Eight samples from the heifers could not be investigated since the cervix was missing. Spermatozoa were observed in 82.5% (45/53) of the examined cervices. One heifer had been mated immediately prior to being harvested (hunter observation), and a high number of spermatozoa were found in the cervix, with an assessed sperm motility of about 60%. In cows (category 5 and 6), the cervix was missing in seven animals, and in the remaining 63 samples, spermatozoa were observed in 53 specimens (84.1%). In a limited number of samples (n = 5), it was clear that mating had taken place approximately 2 weeks into an early pregnancy, since sperm were found in the tract, along with a thin allantochorionic membrane in one of the uterine horns.

In total, 15 heifers and 40 cows were found to be pregnant during the sampling period (day zero to day 103). Pregnancies at varying stages were detected, ranging from thin allanto-chorionic membranes (approximately two weeks) to foetus with a crown-rump length of 276 mm. Pregnant cows were seen from day zero, whereas heifers were seen from day 19. The earliest estimated mating and conception date was mid-September. Of the pregnant heifers (n = 15), eight were yearlings that were sampled in November (Nov 3 – Nov 11) and with a mean weight of 147.4 kg (range 130 – 185 kg).

From all sampled animals (excluding prepubertal heifers and cows with no ovarian activity), 7.6% (15/198) had signs of repeated oestrus, based on the concurrent presence of a regressing corpus luteum together with a mature follicle, or newly developed corpus luteum.

Of the heifers for which an age was determined, the majority (67.4%, 85/126) were yearlings (1.5 years of age) of which 55.3% (47/85) were pre-pubertal. Heifers aged 2.5 (24/126), 3.5 (9/126), 4.5 (7/126), and 6.5 (1/126) years were also identified. The age distribution, pubertal stage, ovulation rates, and status for mating and pregnancy are illustrated in Table 3.

**Table 3:** Categorization of heifers according to findings in 126 sampled moose from southern Sweden

**Prepubertal/Pubertal**	**Age**	**No. of animals**	**Mean carcass weight (kg)**	**Ovulation rate**	**Proportion of mated animals**	**Proportion of pregnant animals**
Prepubertal	1.5	47	122.1	-	-	-
Pubertal	1.5	38	142.6	1.03	0.80	0.21
Prepubertal	2.5	3	161.0	-	-	-
Pubertal	2.5	21	159.7	1.22	0.88	0.19
Prepubertal	3.5	1	150.0	-	-	-
Pubertal	3.5	8	148.3	1.00	0.83	0.13
Pubertal	4.5	7	140.8	1.00	0.80	0.25
Pubertal	6.5	1	145.0	1.00	0	0
All prepubertal	1.5-3.5	51	125.5	-	-	-
All pubertal	1.5-6.5	75	147.3	1.08	0.85	0.23

**Figure 4:**
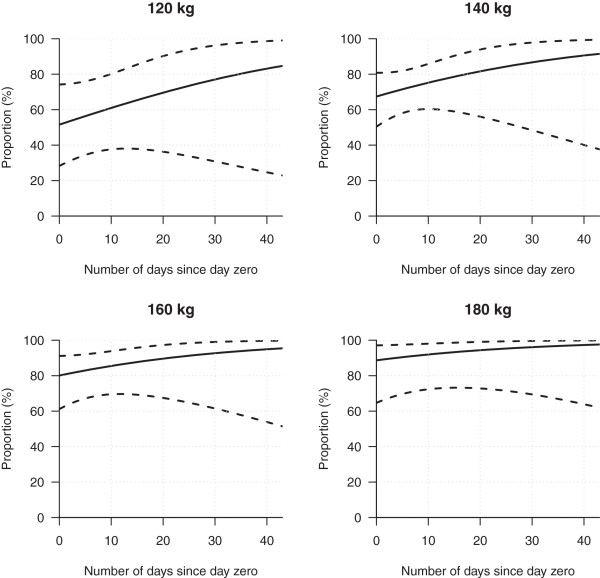
Percentages (with 95% confidence intervals) of female moose (heifers) that passed puberty at four different carcass weight classes.

There was a positive association between the proportion of heifers that had passed puberty, their carcass weight and number of days from day zero (Figure 4). On day zero, approximately 50% of heifers weighing 120 kg had passed puberty, whereas 90% of heifers weighing 180 kg had passed puberty (Figure 4). As the number of days from day zero to harvest increased, the percentage of heifers that passed puberty increased.

**Figure 5:**
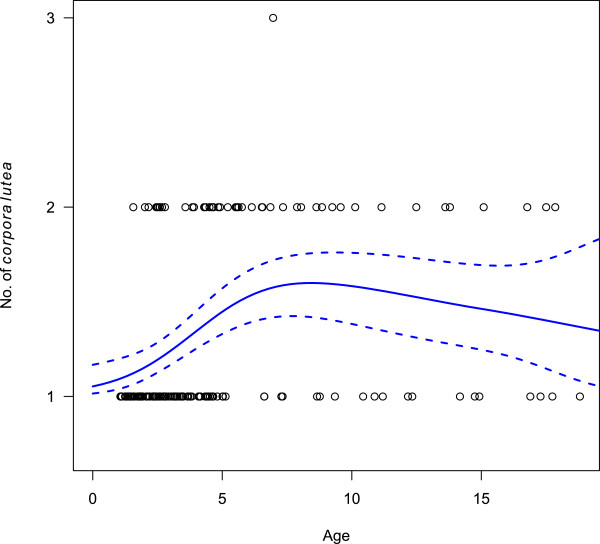
The association between ovulation rates and age of Swedish moose, using logistical regression in a generalized additive model (GAM).

Ovulation rates differed significantly (*P <* 0.01) between heifers (1.08, Table 3), and cows (1.46, n = 107). There was a positive association between body weight and ovulation rate in cows, but no association was found in pubertal heifers. Ovulating females were categorised using the classifications proposed by Ericsson *et al.*[12]: young females (<5 years of age, n = 122), mid-aged females (>5 to 12 years of age, n = 37), and older females (>12 years of age, n = 18). Ovulation rates for the females in these categories were 1.23, 1.54, and 1.39, respectively. The association (with 95% confidence intervals) between ovulation rates and age are illustrated in Figure 5.

## Discussion

The present study provides new information on reproductive characteristics of female moose. The field collection of samples by trained personnel, and the direct handling of the samples in a field laboratory facilitated reliable macroscopic analyses of fresh material. Moose reproductive material degrades quickly following sampling of the animal. These procedures offer an improvement in the quality of samples, and thus the reliability of results, compared to previous studies where samples were transported from the field to the laboratories. As a result, the investigations and assessments of the reproductive organs provided valuable data on puberty, oestrus, ovulation, mating and early pregnancy in moose.

In the present study puberty was used to define when a moose heifer has become capable of reproducing. Saether and Haagenrud [10] reported that the proportion of ovulating yearlings increased with carcass weight. However, non-puberty in older females was not considered.

We show the importance of a high carcass weight to increase the probability of first ovulation/puberty. For instance, the pubertal yearlings had significantly higher mean carcass weights than the pre-pubertal ones. A delay in the onset of puberty may be attributed to the lasting effect of a low calf body weight [26], and/or with the quality/availability of summer forage, which in turn may be dependent on the density of moose and other cervids. Sand [9] reported similar findings, but on an age-dependent spatial scale. Surprisingly, the present study showed that some females, from 3.5 up to 6.5 years of age, were still heifers (i.e. there were no corpora albicantia in the ovaries). It is likely that these “over-aged” heifers had been overlooked in previous studies of sexual maturity (defined as a female having produced offspring) in moose.

Another important aspect of puberty in the present study was that the proportion of heifers that had experienced their first oestrus was also positively correlated with time of season (see below). If mated late during the season, this affects the time of calving for heifers the subsequent year, and may also explain lower carcass weights in calves harvested from young females [11,27].

Using the estimated time of mating of the pregnant animals and the presence of large corpora lutea in the ovaries, the results from the present study indicate that the peak of oestrus (first oestrus of the season) and ovulation occurs before the start of the hunting period in southern Sweden. The time period during which females have their first oestrus of the season is most likely longer than was previously reported. Broberg [8] assumed that the breeding period of moose in Sweden occurs during the last week of September and the beginning of October, which was in accordance with the findings of Markgren [4]. In both studies, the calculated time of oestrus was based on the subtraction of a gestation period of fixed length (234 days, [4]) from the date of the birth of the offspring. However, according to Schwartz and Hundertmark [28] the gestation period of moose varies from 214 up to 240 days, which needs to be considered. Calculating from the calving date, using a fixed gestation period (234 days), or not taking into consideration that the breeding period in a moose population is longer than two to three weeks, may lead to the conclusion that a significant proportion of females are fertilized at their second oestrus of the season as was done by Broberg [8]. The present study found that a low proportion of females had a second oestrus, based on the findings of regressed corpora lutea in the ovaries. However, we also found some animals that passed oestrus but had no sperm in their cervices. If not harvested, these females would probably have returned to oestrus.

Absence of mating in some animals, as shown in the present study (no findings of spermatozoa) could be due to a shortage of available males, as bulls of all ages are hunted from day zero and which may alter the bull/cow ratio. Another reason may be that the courting behaviour and subsequent mating was disturbed by hunting activities, such as the use of hunting dogs. Neumann [29] reported a marked change in moose movement behaviour when hunting dogs were in the area.

This study found that the oestrus period for heifers generally occurred later in the season than for cows, which is in accordance with Haagenrud and Markgren [11]. Previous Fennoscandian moose studies have calculated a temporal and spatial variation in the timing of oestrus [5,11], but no such variation (between areas or years) was observed in the present study, which downplays the effect of the surrounding environment on timing of oestrus in the present study.

The present study demonstrated that the oestrus period for moose has not finished when the hunting period starts. The effects of this overlap on moose reproductive success is not known, but needs to be studied further. In the 1980’s, a mutual agreement was reached among hunting stakeholders in Sweden (government authorities and hunters’ associations) that hunting activities should not overlap or interfere with breeding period in moose (Pettersson, personal communication). The results from the present study suggest that, if such an agreement was intended as a guideline for the timing of the hunting season, a revision of the hunting period for moose, at least in southern Sweden, may be required. In addition, similar studies regarding the timing of oestrus in mid- and northern Sweden would be beneficial in order to determine if a hunting period/oestrus overlap is present also in those areas.

Regarding the time of ovulation in relation to oestrus, we still have limited information, based on the investigations in the present study, on whether ovulation occurs during, or after standing oestrus. A hunter observed one of the sampled heifers being mated approximately two hours before the heifer was harvested. Macroscopic examination of the ovaries from this animal showed a newly erupted follicle, i.e. ovulation had occurred. Thus, we cannot conclude if the ovulation occurred during the standing oestrus or just after. According to Schwartz and Hundertmark [28], the duration of standing oestrus is short and varies from one to 36 hours in moose.

Ovulation rates have been used as a measure of fecundity in moose [30-32], but this is not an accurate measure of fertility. The concept of fertility includes the ability to produce offspring, which cannot be determined solely by the investigation of ovarian activity. Also, ova loss and embryonic mortality may affect fertility [33]. Nevertheless, fecundity and fertility may be related, as previous reports on fecundity in Swedish moose indicate that ovulation rates [30] and calf numbers [15] among young and old-aged moose are lower than in mid-aged moose.

## Conclusions

In conclusion, the investigation of fresh and intact reproductive organs for studies of reproduction in wildlife provides valuable information. Puberty (first oestrus and ovulation) in female moose, regardless of age, appears to be related to body weight and time of the breeding season. In addition, the further into the hunting period in the autumn, a higher proportion of heifers reach puberty. The detection of spermatozoa in the cervical mucosa was a useful method to detect the occurrence of mating. Female moose show oestrus during a longer period of the season than previously reported, and in some animals (mostly heifers), the hunting period coincided with oestrus. From these findings, it is therefore reasonable to consider that the hunting period should be postponed by at least 14 days in southern Sweden to avoid an overlap between the hunting period and oestrus in moose.

## Competing interests

The authors declare that they have no competing interests.

## Authors’ contributions

JM planned and designed the study, took part in all fieldwork (sample collection) and lab work, completed the data analysis and drafted the original manuscript. AMD had the main responsibility for the study, took part in all fieldwork and lab work, assisted in the data analysis, and contributed to the manuscript. LS was partly responsible for the study, assisted in the study design, assisted during part of the field work and the data analysis, and contributed to the manuscript. CGT assisted in the study design, assisted during part of the fieldwork and the data analysis, and contributed to the manuscript. DGW was responsible for the Swedish part of the Wildtech project, and contributed to the manuscript. LY participated in the Wildtech project, and contributed to the manuscript. MRH participated in the Wildtech project, and contributed to the manuscript. All authors have read and approved the final version of the manuscript.
